# Does dysbiotic endometrium affect blastocyst implantation in IVF patients?

**DOI:** 10.1007/s10815-019-01630-7

**Published:** 2019-11-18

**Authors:** Tomoko Hashimoto, Koichi Kyono

**Affiliations:** Kyono ART Clinic Takanawa, Takanawa Court 5F 3-13-1, Takanawa, Minato-ku, Tokyo, 108-0074 Japan

**Keywords:** Endometrium, Microbiota, High-throughput nucleotide sequencing, Blastocyst, Embryo implantation

## Abstract

**Purpose:**

To analyze the pregnancy outcomes of IVF patients presenting eubiotic or dysbiotic endometrium at the time of embryo transfer and to analyze what bacterial profiles are suitable for embryo implantation.

**Methods:**

Ninety-nine IVF patients under 40 years old undergoing vitrified-warmed blastocyst transfer in HRT cycle had concurrent endometrial microbiome analysis. Samples from the endometrium were taken from the participants at the time of mock transfer; the bacterial profiles at genus level and percentage of lactobacilli in the endometrium of the patients were analyzed.

**Results:**

Thirty-one cases (31.3%) had dysbiotic endometrium. The background profiles, pregnancy rates per transfer (52.9% vs 54.8%), and miscarriage rates (11.1% vs 5.9%) were comparable between patients with eubiotic or dysbiotic endometrium. Major bacterial genera other than *Lactobacillus* detected in the dysbiotic endometrium were *Atopobium*, *Gardnerella*, and *Streptococcus*. Some patients achieved ongoing pregnancies with 0% *Lactobacillus* in the endometrium. The endometrial bacterial profiles of pregnant cases with dysbiotic endometrium were comparable with those of non-pregnant cases.

**Conclusion:**

Analyzing microbiota at the species-level resolution may be necessary for identifying the true pathogenic bacteria of the endometrium and avoiding over-intervention against non-*Lactobacillus* microbiota. Further studies are necessary for analyzing the mechanism of how the pathogenic bacteria affect embryo implantation.

**Electronic supplementary material:**

The online version of this article (10.1007/s10815-019-01630-7) contains supplementary material, which is available to authorized users.

## Introduction

Recent studies using next-generation sequencing of the 16S rRNA gene revealed the existence of an endometrial microbiota represented by *Lactobacillus* and other bacteria [1, 2]. Also, recent reports have suggested that human uterine microbiota is related to implantation success and that non-*Lactobacillus*-dominated microbiota (NLDM), defined as < 90% *Lactobacillus* spp., was associated with significant decrease in implantation, pregnancy, ongoing pregnancy, and live birth rates [3]. Meanwhile, in our previous study, pregnancy rate of IVF was higher in patients with ≥ 80% *Lactobacillus*-dominated endometrial microbiota compared to that with < 80% *Lactobacillus* [4], and *Bifidobacterium*-dominant endometrium was suspected to be an acceptable environment for implantation [4]. On the other hand, we also experienced a few cases who achieved pregnancy in spite of the non-*Lactobacillus*-dominated endometrial status [4].

To what extent the human uterine microbiomes are involved in implantation failure, and whether or not dysbiotic endometrium really have an impact on embryo implantation, is still not clear. This present study aimed to analyze the pregnancy outcomes of IVF patients presenting eubiotic or dysbiotic endometrium at the time of embryo transfer and to analyze what bacterial profiles are suitable for embryo implantation.

## Materials and methods

### Patients and samples

A total of 116 IVF patients under 40 years old undergoing vitrified-warmed blastocyst transfer (FBT) agreed to undergo simultaneous endometrial microbiome analysis in our center from February 2019 to August 2019. Among the 116 patients, patients with (1) severe male factor, such as severe oligozoospermia, cryptozoospermia, or azoospermia; (2) hydrosalpinx; (3) uterine malformation; (4) submucosal myoma; or (5) antibiotic usage within a month prior to embryo transfer were excluded from this study. During consultation with the doctor, the participants had no complaints suggestive of vaginitis or endometritis. Finally, 99 patients were eligible for this study.

FBT was performed in a hormonal replacement therapy (HRT) cycle. After appropriate priming of estradiol (either by estradiol transdermal patch or estradiol valerate or both when necessary) for about 1–2 weeks leading to a trilaminar endometrium of ≥ 6 mm and confirming appropriate hormonal status, progesterone (P) was administered, either by micronized progesterone suppositories or by chlormadinone acetate for 5 full days, and on day P + 5, blastocyst transfer was performed. For patients with a history of ERA (Igenomix, Spain), blastocyst transfer was performed according to the designated timing, such as P + 6 [5]. All patients were routinely examined by vaginal ultrasound with a sterilized probe cover to confirm the direction of the uterus and uterine cavity length prior to embryo transfer. The last follow-up date was September 2, 2019.

This study was approved by the Institutional Review Board of Kyono ART Clinic Takanawa on July 29, 2017. All the patients involved in this study have allowed us to use their medical record data for research in an unidentifiable manner. Written informed consent was obtained from all patients prior to sample collection.

### Sample collection and microbiome analysis

Samples from the endometrium were taken from the participants at the time of mock transfer. We routinely perform mock transfer using an IUI catheter in our clinic. After cleansing of the mucous around the cervical os and the uterine cervix with sterile saline, the cleansed saline solution was thoroughly aspirated with a sterilized syringe; after the cervical mucous was again carefully aspirated with another sterilized syringe to avoid contamination, endometrial fluid (EF) specimens were carefully aspirated with a Kitazato IUI catheter (Kitazato Corporation, Japan) with utmost care not to touch the vaginal wall [6]. These were sterilely put into a 1-mL MMB collection tube (DNA Genotek Inc., Canada) and were sent to Varinos Inc., Japan, for microbiome analysis. The bacterial profiles at genus level, percentage of lactobacilli in the endometrium of the patients, were provided by the endometrial flora test (Varinos Inc., Japan) [6]. There was no bleeding induced by EF aspiration. After the samples were collected by the mock ET catheter, FBT was performed.

### DNA extraction, polymerase chain reaction (PCR) amplification, and DNA sequencing

Endometrial samples were treated with proteinase K and lysozyme solution according to the manufacturer’s instructions. Genomic DNA was extracted using Agencourt Genfind v2 Blood & Serum DNA Isolation Kit (Beckman Coulter Inc., USA). dsDNA concentration was quantified fluorometrically with a Qubit dsDNA HS Assay Kit (Thermo Fisher Scientific Inc., USA).

The variable region 4 (V4) hypervariable region of the bacterial 16S rRNA gene was amplified from the specimen’s DNA using a modified primer pair 515f (5′ - TCGTCGGCAGCGTCAGATGTGTATAAGAGACAGGTGYCAGCMGCCGCGGTAA - 3′) and 806rB (5′ - GTCTCGTGGGCTCGGAGATGTGTATAAGAGACAGGGACTACNVGGGTWTCTAAT - 3′), with Illumina Nextera XT adapter overhang sequences (underlined) [7]. Universal bacterial primers to amplify the V1–V2 region and V3–V5 regions were 28f (5′ - TCGTCGGCAGCGTCAGATGTGTATAAGAGACAGGAGTTTGATCNTGGCTCAG - 3′) to 338r (5′ - GTCTCGTGGGCTCGGAGATGTGTATAAGAGACAGTGCTGCCTCCCGTAGGAGT - 3′) and 357f (5′ - TCGTCGGCAGCGTCAGATGTGTATAAGAGACAGCCTACGGGAGGCAGCAG - 3′) to 926r (5′ - GTCTCGTGGGCTCGGAGATGTGTATAAGAGACAGCCGTCAATTYMTTTRAGT - 3′), respectively. PCR amplification was performed as previously described [3]. PCR was performed with 25 ng/μL DNA, 200 μmol/L of each of the 4 deoxynucleotide triphosphates, 400 nmol/L of each primer, 2.5 U of FastStart HiFi polymerase, 4% of 20 mg/mL BSA (Sigma), 0.5 mol/L betaine (Sigma), and the appropriate buffer with MgCl2 supplied by the manufacturer (Roche). Thermal cycling consisted of initial denaturation at 94 °C for 2 min followed by 30 cycles of denaturation at 94 °C for 20 s, annealing at 50 °C for 30 s, extension at 72 °C for 1 min, and final extension at 72 °C for 5 min. Amplicon mixture was purified using Agencourt AMPure XP (Beckman Coulter Inc., USA). Purified PCR samples were multiplexed using a dual-index approach with the Nextera XT Index kit v2 (Illumina Inc., USA) according to the Illumina 16S Metagenomic Sequencing Library Preparation protocol. Indexing PCR was performed with KAPA HiFi HotStart ReadyMix (Kapa Biosystems) in a 50-μL reaction volume, and subsequently, purification was performed with Agencourt AMPure XP beads. The final library was paired-end sequenced at 2 × 200-bp or 2 × 300-bp using a MiSeq Reagent Kit v3 on the Illumina MiSeq platform depending on primer set. Since the 16S rRNA gene target region had an immense impact on analysis results, we compared V1–V2, V3–V5, and V4 variable regions commonly used in 16S rRNA sequencing from human samples [8]. To evaluate the representation of the microbial community, we used the ZymoBIOMICS Microbial Community Standard (Zymo Research, USA) containing a mixture of *Pseudomonas*, *Escherichia*, *Salmonella*, *Lactobacillus*, *Enterococcus*, *Listeria*, *Bacillus*, and two yeast species. All species except *Salmonella* were observed by all primer sets; however, V1–V2 and V3–V5 amplicon sequencing failed to detect *Salmonella* (data not shown). To evaluate whether some target regions better represented endometrial microbial community structure than other regions, three variable regions of 10 endometrial samples were sequenced. Detection of *Gardnerella* and *Bifidobacterium* were observed in V3–V5 and V4 amplicon sequencing but not in V1–V2. From these results, primer set targeting the V4 region was used for the endometrial microbiome analysis.

### Data analysis

Reads were merged using EA-Utils fastq-join [9] and a median merged sequence length of 291 bp was obtained. Quality control for merged sequences was performed using USEARCH v10.0.240 [10] to remove PhiX reads, truncate primer-binding sequences, and discard sequences with < 100 bp length and sequence quality < Q20. QIIME 1.9.1 [11] was used with default parameters for quality filtering, chimera check, clustering sequences into OTUs, and assignment of taxonomy. Sequences were clustered into OTUs by open-reference OTU picking strategy using the UCLUST method based on 97% sequence identity. Taxonomy was assigned to each OTU using the RDP classifier [12] with 0.50 confidence threshold against the Greengenes database version 13_8 [13]. Low-abundance taxa (0.01%) were filtered from the OTU tables. All further analyses were performed at a rarefied depth of 5000 sequences per sample to correct for differences in read depth across samples. Since human specimens contain low bacterial DNA content, background bacterial contamination critically affected the result [14]. If the library concentration of an endometrial sample was as much as blank control, UltraPure™ DNase/RNase-Free Distilled Water (Thermo Fisher Scientific Inc., USA), the result of the microbial community was similar to the blank control. This similarity to the background microbiome makes it difficult to determine presence of unique endometrial taxa; therefore, blank-characteristic OTUs were subtracted to reduce background noise, as in previous studies [15, 16]. The following 9 bacterial taxa found in a blank control and known as reagent contaminations were excluded from endometrial samples using QIIME: *Acinetobacter*, *Escherichia*, *Flavobacterium*, *Janthinobacterium*, *Methylobacterium*, *Pseudomonas*, *Rhodococcus*, *Sphingomonas*, and *Stenotrophomonas*.

The patient profiles, bacterial status, percentage of lactobacilli in endometrium of the patients, and pregnancy outcomes were analyzed. Clinical pregnancy was defined as confirmed gestational sac in the uterine cavity by ultrasound analysis. Ongoing pregnancy was defined as the pregnancy having completed ≥ 12 weeks gestation.

### Statistical analysis

Statistical analysis (using the StatMate V software (Tokyo, Japan)) was performed by using *t* test, Mann–Whitney *U* test, chi-square analysis, or Fisher’s extract test where appropriate. A *P* value of less than 0.05 was considered statistically significant.

## Results

### Patient profiles

The baseline characteristics of the 99 IVF patients were as follows. Average age was 35.26 ± 2.98 years old; BMI, 20.39 ± 2.26; multigravida, 51 cases (51.5%); and multipara, 29 cases (29.3%). All cases were Japanese. The past histories of failed embryo transfers were 1.20 ± 1.77 cycles.

Considering the results from our previous study [4], we defined a eubiotic endometrium as ≥ 80% *Lactobacillus* + *Bifidobacterium* spp. (eubiosis) and a dysbiotic endometrium as < 80% *Lactobacillus* + *Bifidobacterium* spp. with ≥ 20% of other bacteria (dysbiosis). Using this criteria, 68 cases (68.7%) were eubiosis and 31 cases (31.3%) were dysbiosis.

There were no significant differences in age, BMI, serum anti-Müllerian hormone (AMH) level, duration of infertility, numbers of previous failed transfer cycles, gravidity, and parity between the eubiosis and dysbiosis (Table 1). A total of 17 patients (12 in eubiosis and 5 in dysbiosis) underwent ERA testing prior to the microbial analysis (Table 1). Among these, 6 patients in eubiosis (50%) and 2 patients in dysbiosis (40%) had altered transfer timing designated by ERA, which was comparable between the both groups.

**Table 1:** Background of the two groups

	Eubiosis	Dysbiosis	*P* value
No. of patients	68	31	–
Age (years): mean ± SD	35.19 ± 3.04	35.42 ± 2.87	NS
BMI: mean ± SD	20.56 ± 2.29	20.03 ± 2.18	NS
Serum AMH (ng/ml): mean ± SD	5.44 ± 7.70	4.14 ± 3.16	NS
Duration of infertility (months): mean ± SD	29.72 ± 22.46	29.61 ± 28.36	NS
Previous ET: mean ± SD	0.97 ± 1.54	1.71 ± 2.13	NS
Multigravida patients: *N* (%)	33 (48.5)	18 (58.1)	NS
Multipara patients: *N* (%)	19 (27.9)	10 (32.3)	NS
Patients with endometriosis: *N* (%)	2 (2.9)	2 (6.5)	NS
Patients with myoma: *N* (%)	2 (2.9)	2 (6.5)	NS
Patients with PCOS: *N* (%)	16 (23.5)	6 (19.4)	NS
Patients with ERA performed: *N* (%)	12 (17.6)	5 (16.1)	NS
% of endometrial LB: median (range)	98.65 (0–100)	15.10 (0–78.60)	< 0.001

*AMH* anti-Müllerian hormone, *ET* embryo transfer, *LB Lactobacillus*, *PCOS* polycystic ovary syndrome, *ERA* endometrial receptivity analysis

A total of 13,598,805 sequence reads were obtained with a mean 137,362 reads per sample (range 30,037–378,870) in EF. The median percentage of endometrial lactobacilli in eubiosis and dysbiosis were 98.65% (0–100) and 15.10% (0–78.60), respectively (*P* < 0.001, Mann–Whitney test) (Table 1).

### Pregnancy outcome of patients: eubiotic vs dysbiotic endometrium

Single vitrified-warmed blastocyst transfers were performed in all cases except one double-blastocyst transfer in the dysbiosis group. All transfers were performed under transabdominal ultrasound guidance. The morphological quality of the blastocysts was measured according to Gardner’s criteria [17], and good-quality blastocysts were defined as grade 3BB or better on day 5 or day 6. In our clinic, we always vitrify blastocysts graded 3BC or better. Quarter zona pellucida opening by laser-assisted hatching (AHA) was performed with some modifications [18] depending on the patient’s request. The percentage of morphologically good-quality blastocysts transferred and the percentage of laser AHA performed were comparable between the two groups (Table 2).

**Table 2:** Characteristics and pregnancy outcome of two groups (eubiosis vs dysbiosis)

	Eubiosis	Dysbiosis	*P* value
No. of patients	68	31	–
No. of FBT	68	31	–
No. of transferred blastocysts	68	32	–
Good-quality blastocysts transferred: *N* (%)	59 (86.8)	26 (81.3)	NS
Laser AHA performed: *N* (%)	49 (72.1)	25 (78.1)	NS
No. of pregnancies	36	17	–
No. of ongoing pregnancies^a^	26	14	–
No. of miscarriages^a^	4	1	–
No. of twin pregnancies	0	1	–
No. of ectopic pregnancies	0	0	–
Pregnancy rate (per FBT) (%)	52.9	54.8	NS
Implantation rate (No. of GS/transferred blastocysts) (%)	52.9	53.1	NS
Miscarriage rate (per pregnancy) (%)	11.1	5.9	NS

Ongoing pregnancy was defined as the pregnancy having completed ≥ 12 weeks gestation

*FBT* frozen-thawed blastocyst transfer, *AHA* assisted hatching, *GS* gestational sac

^a^As of September 2, 2019

Pregnancy rates per FBT (52.9% vs 54.8%), implantation rates (52.9% vs 53.1%), and miscarriage rates (11.1% vs 5.9%) were comparable between eubiosis and dysbiosis, respectively (Table 2).

### Endometrial microbial communities of pregnant and non-pregnant cases with dysbiotic endometrium

**Fig. 1:**
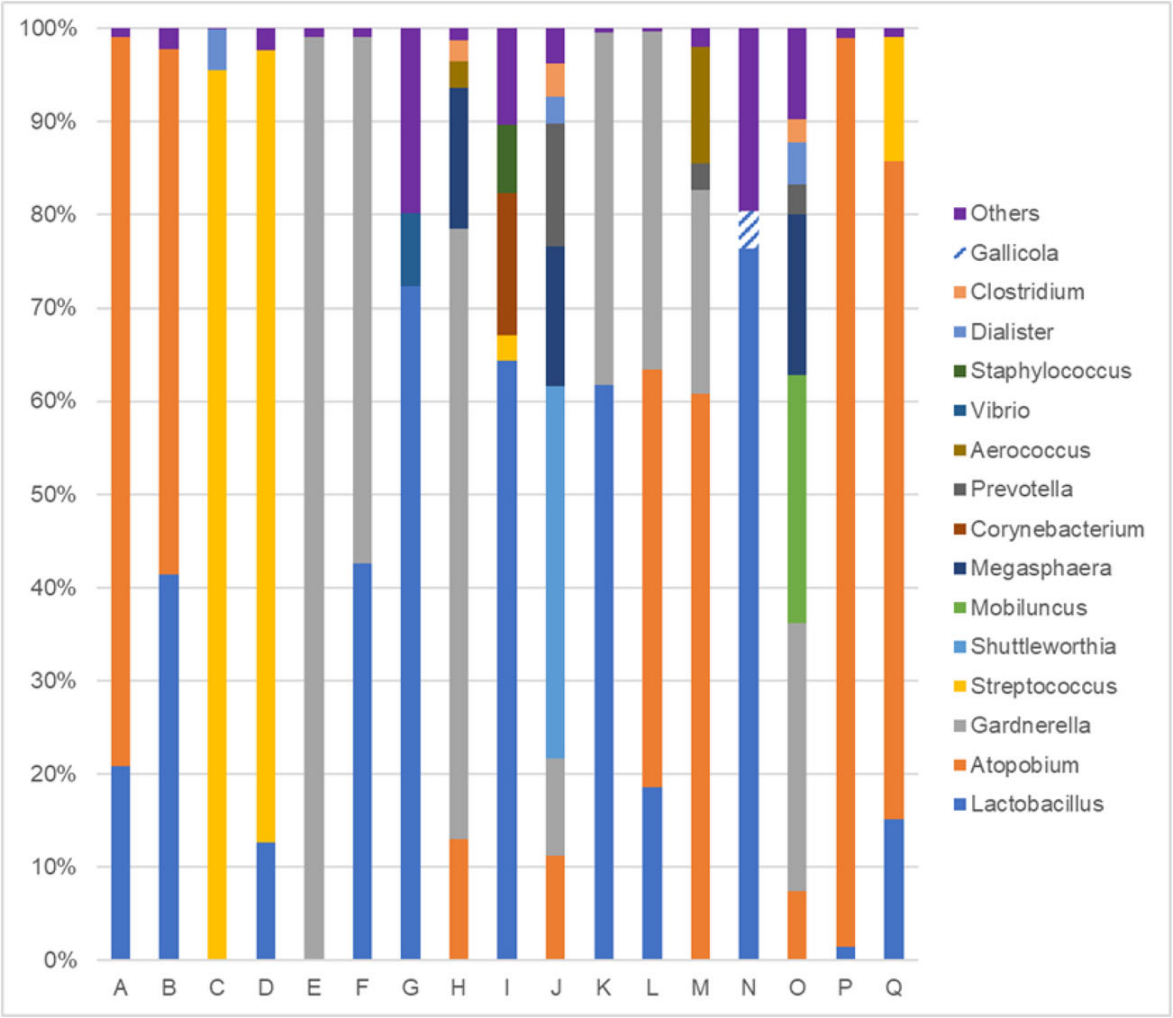
Bacterial profile of patients with dysbiotic endometrium achieving FBT pregnancy. The letters below the graph correspond to individual patients. Patients A to N have ongoing pregnancies beyond 15 weeks as of September 2, 2019. Patient Q had spontaneous miscarriage at 6 weeks

Seventeen patients achieved FBT pregnancy in dysbiosis (Fig. 1). The median percentage of endometrial lactobacilli in those patients was 15.1% (range 0–76.4%). Major bacterial genera detected were *Atopobium* (7.3–97.4%), *Gardnerella* (10.5–98.9%), and *Streptococcus* (2.7–95.5%) (Fig. 1).

**Fig. 2:**
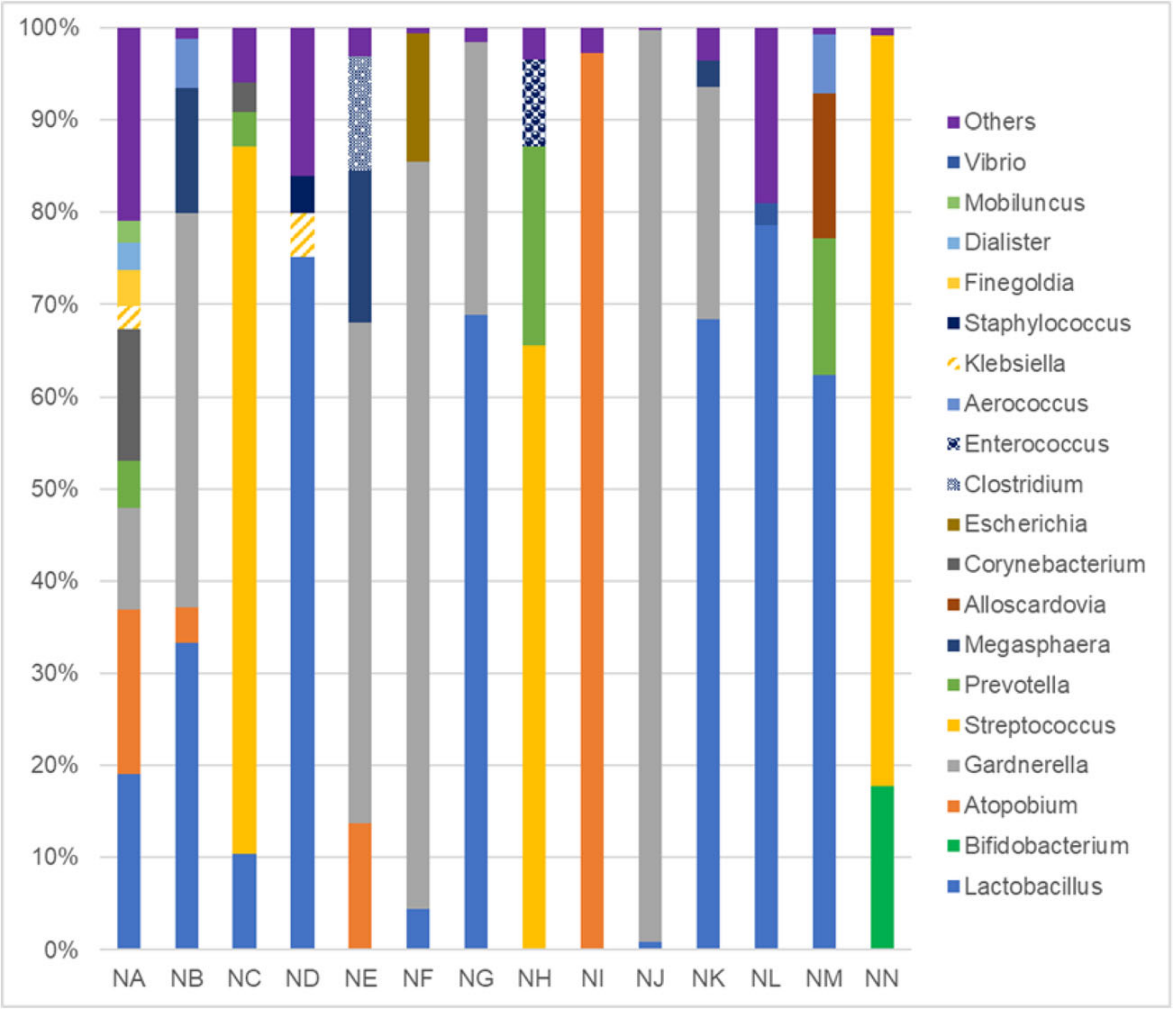
Bacterial profile of patients with dysbiotic endometrium who did not achieve FBT pregnancy. The letters below the graph correspond to individual patients

Meanwhile, 14 patients did not achieve FBT pregnancy. The median percentage of endometrial lactobacilli in these patients was 14.75% (0–78.6%). Major bacterial genera detected in these patients were *Gardnerella* (11.0–98.8%), *Atopobium* (3.8–97.3%), and *Streptococcus* (65.4–81.5%), which was comparable to the bacterial status of pregnant cases with dysbiotic endometrium (Fig. 2). There was no significant difference in the background of the pregnant and non-pregnant cases with dysbiotic endometrium (Table 3).

**Table 3:** Background of the pregnant and non-pregnant patients in dysbiosis

	Pregnant	Non-pregnant	*P* value
No. of patients	17	14	–
No. of FBT	17	14	–
Age (years): mean ± SD	34.71 ± 3.08	36.29 ± 2.43	NS
BMI: mean ± SD	19.8 ± 1.78	20.31 ± 2.62	NS
Serum AMH (ng/mL): mean ± SD	4.63 ± 3.39	3.54 ± 2.85	NS
Duration of infertility (months): mean ± SD	34 ± 29.34	24.29 ± 27.21	NS
Previous ET: mean ± SD	1.82 ± 2.35	1.57 ± 1.91	NS
Multigravida patients: *N* (%)	7 (41.2)	11 (78.6)	NS
Multipara patients: *N* (%)	4 (23.5)	6 (42.9)	NS
No. of transferred blastocysts	18	14	–
Good-quality blastocysts transferred: *N* (%)	15 (83.3)	11 (78.6)	NS
% of endometrial LB: median (range)	15.10 (0–76.40)	14.75 (0–78.60)	NS

*AMH* anti-Müllerian hormone, *FBT* frozen-thawed blastocyst transfer, *ET* embryo transfer, *LB Lactobacillus*

## Discussion

This may be the first report in Japan analyzing endometrial microbiota concurrently at the time of embryo transfer and analyzing the direct impact of uterine microbial environment on pregnancy. Previous reports, including our previous study, indicated that *Lactobacillus* dominancy in the endometrium was favorable in terms of pregnancy outcome [3, 4]. Moreno et al. reported that the adverse effect of NLDM on pregnancy was more evident in subjects presenting dominant *Gardnerella* and *Streptococcus* genera [3]. Also, bacterial genera such as *Enterococcus*, *Enterobacteriaceae*, *Streptococcus*, *Staphylococcus*, *Gardnerella*, *Mycoplasma*, *Ureaplasma*, *Chlamydia*, and *Neisseria* are reported to be responsible for chronic endometritis (CE) [19] and are suspected to adversely affect implantation. But in the present study, embryo implantation was not affected by the non-*Lactobacillus*-dominated endometrium, and *Atopobium*, *Gardnerella*, and *Streptococcus* dominancy was acceptable for implantation in a subset of patients (Table 2, Fig. 1). There were patients who achieved FBT pregnancies with 0% *Lactobacillus* and 95.5% *Streptococcus*, or 0% *Lactobacillus*, 60.8% *Atopobium*, and 21.9% *Gardnerella* (Fig. 1); these patients currently have ongoing pregnancies beyond 16 weeks. Meanwhile, there were patients who could not achieve FBT pregnancy with 0% *Lactobacillus* and 97.3% *Atopobium*, or 0% *Lactobacillus*, 54.3% *Gardnerella*, and 13.7% *Atopobium* (Fig. 2). Patient NH (Fig. 2) was pathologically diagnosed as CE 1 month after the microbial analysis; *Streptococcus* was dominantly detected at the time of initial transfer (Fig. 2) and thus suspected to be the possible pathogen of CE and one of the causes of implantation failure. As the number of cases was limited in this study, we cannot conclude from these inconsistent results, but some of the bacteria other than *Lactobacillus* or *Bifidobacterium* spp. detected in the uterine cavity may be simply residents, not pathogens, of the upper female reproductive tract [20]. Pathogenicity may differ by bacterial species; *Streptococcus agalactiae* and *Streptococcus anginosus* are classified in the same bacterial genus but may act differently in the endometrium. The potential regulatory mechanisms of each microbial species on implantation are still not clear, and currently, we can only speculate from the previous findings regarding chronic endometritis or bacterial vaginosis, etc. It may be speculated that NLDM may trigger an inflammatory response in the endometrium that affects embryo implantation, as inflammatory mediators are tightly regulated during the adhesion of the blastocyst to the endometrial epitherium [3]. *Streptococcus agalactiae* is regarded as one of the major pathogens of CE [19] and is also well-known as one of the leading causes of neonatal infections by vertical transmission from colonized mothers. CE has been suggested to contribute to diminished success rates of both spontaneous and IVF conceptions as well as obstetrical/neonatal complications [19]. *Atopobium vaginae* and *Gardnerella vaginalis* are known to be the major bacterial vaginosis-associated bacteria; they stimulate an innate immune response from vaginal epithelial cells and possibly contribute to the pathogenesis of bacterial vaginosis [21]. *Bifidobacterium* is the dominant member of some vaginal microbiomes and is suggested to have the potential to be as protective as lactobacilli, contributing to a healthy vaginal microbiota in reproductive aged women [22]. *Lactobacillus* spp.-dominated human vagina has been known to be protective against cervico-vaginal infections; however, the level of protection against infection varies by species or strain of *Lactobacillus*, and some species, although dominant, are not always optimal [23]. This may arise from the fact that antimicrobial factor (e.g., lactic acid) producing ability differs with each *Lactobacillus* spp. [24, 25]. In that sense, analyzing microbiota at the species-level resolution may be necessary for identifying the true pathogenic bacteria of the endometrium and avoiding over-intervention against non-*Lactobacillus* microbiota; further studies are necessary to analyze the mechanism of how the pathogenic bacteria affect embryo implantation. Furthermore, not only the existence of pathogens but also the immune status of the host itself may be more critical for presenting clinical manifestations [26, 27].

This study is different from the previous study [3] in terms of background (ethnicity, age, etc.), transferred embryos, definition of eubiosis/dysbiosis, and the timing of sample collection; those factors may have contributed to the different research outcome of this present study. As for definition, Moreno et al. defined the bacterial status of the endometrium as *Lactobacillus*-dominated microbiota (LDM, > 90% *Lactobacillus* spp.) or non-*Lactobacillus*-dominated microbiota (NLDM, < 90% *Lactobacillus* spp. with > 10% of other bacteria), based on the composition of the microbiota in the endometrial fluid [3], and reported that the presence of NLDM was associated with significant decrease in implantation, pregnancy, ongoing pregnancy, and live birth rates [3]. Meanwhile, in our previous study, the pregnancy rate of IVF was comparable between LDM and NLDM; however, it tended to be higher in patients with ≥ 80% *Lactobacillus*-dominated endometrial microbiota compared to those with < 80% *Lactobacillus* [4]; furthermore, *Bifidobacterium*-dominated endometrium was also suspected to be an acceptable environment for implantation [4]. Thus, in the current study, we defined a eubiotic endometrium as ≥ 80% *Lactobacillus* + *Bifidobacterium* spp. (eubiosis) and a dysbiotic endometrium as < 80% *Lactobacillus* + *Bifidobacterium* spp. with ≥ 20% of other bacteria (dysbiosis). Actually, we have analyzed our current data according to the previous criterion of LDM vs NLDM [3] and found comparable implantation/pregnancy/miscarriage rates between LDM vs NLDM (Supplementary tables 1 and 2).

Considering the background of this study, the participants included in this study were relatively young and were not necessarily limited to recurrent implantation failure (RIF). Bacterial profiles and the pregnancy outcomes may have been different if the subjects had been limited to RIF patients. This study included only Japanese infertile patients and the results may have been different with people of other ethnicities. Preimplantation genetic testing for aneuploidy (PGT-A) or oocyte donations are prohibited in Japan at present and were therefore not performed in this study. If PGT-A had been used in this study, patient recruitment and outcomes may have been different, because PGT-A would have influenced embryo selection; but the patient profiles and the quality of transferred blastocysts were comparable between eubiosis and dysbiosis (Tables 1 and 2).

As for the timing of sample collection, previous studies had microbial analysis in cycles before embryo transfer [3, 4]; but there are possibilities that microbiomes change over time, so we consider sampling timing as the critical factor of the study. Franasiak et al. [1] analyzed endometrial microbiome from the transfer catheter of 33 patients undergoing euploid single embryo transfer and compared the bacterial profiles between patients with ongoing pregnancy vs those without ongoing pregnancy; *Lactobacillus* was the top species call for both outcomes, and there were major species which appeared to vary by outcome, but the differences were not statistically significant [1]. In our present study, the EF specimens were collected immediately before embryo transfer; thus, the microbial results were assumed to reflect the environment of the uterine cavity during the window of implantation among Japanese infertile patients. There may be a concern that EF specimens may have contained some endocervical fluid at the time of sample collection, or the transvaginal examination may have influenced the endometrial microbial results, thus limiting this study. Using a double-lumen catheter for EF aspiration [3] may avoid the contamination of endocervical fluid; however, the cost for such a device was much more expensive compared to an IUI catheter as used in our study. Considering that the uterine cavity and the cervical canal is a continuum and that vaginal and endometrial bacterial communities are reported to be closely related in most of the subjects tested [3], it can be assumed that the microbial results obtained in this study mostly reflected the microbial environment of the uterine cavity. EF can be aspirated in the same cycle as embryo transfer is performed without negatively affecting implantation [28, 29]; thus, EF aspiration prior to embryo transfer in our present study was suspected to be harmless for implantation. Meanwhile, microbiota composition in EF may not fully reflect that in endometrial tissue [30]; however, endometrial tissue sampling is harmful when performed immediately before embryo transfer. The exact amount of each aspirated EF was not measured in our study, but the quality of the samples was sufficient for microbiome analysis with a library concentration > 10 ng/μL.

Other limitations of this present study are the short follow-up period, limited study numbers, absence of analysis of other gynecological histories (e.g., bacterial vaginosis), lifestyle habits (such as sexual contact and sanitary conditions), past oral contraceptive usage, etc. All the EF samples in this study were collected in an HRT cycle. There may be a concern that hormonal therapy may have influenced on endometrial microbiome, but the hormonal regimen in this study was mostly uniform, and it is reported that endometrial microbiota is independent of hormonal regulation [3]. To the best of our knowledge, there are no reports analyzing the relationship between endometrial microbiome and hormonal therapy; there are also no reports comparing the route of estrogen delivery and degree of change in the vaginal microbial community [31].

As there were a small number of patients who had a miscarriage during this study period, the correlation between endometrial microbiota and miscarriage was not analyzed. There might be a correlation between miscarriage or preterm birth and dysbiotic endometrium at the time of embryo transfer, but this remains to be elucidated.

Antibiotic resistance is a growing problem and there is a risk of disturbing normal bacterial flora with the blind, cumulative usage of antibiotics. Not every single microbe other than *Lactobacillus* spp. may necessarily be eradicated from the endometrium. In order to avoid over-intervention with antibiotics, further studies are necessary to properly diagnose a “true dysbiosis” of the endometrium resulting in implantation failure.

## Electronic supplementary material

Supplementary Table 1 (DOCX 17 kb)

Supplementary Table 2 (DOCX 17 kb)
