# Supplementary material for: Does dysbiotic endometrium affect blastocyst implantation in IVF patients?
Source: J Assist Reprod Genet. 2019 Nov 18;36(12):2471–9. doi: 10.1007/s10815-019-01630-7 (PMC6910901; doi:10.1007/s10815-019-01630-7)
Supplement: Supplementary file 1 — (DOCX 17 kb) [file 10815_2019_1630_MOESM1_ESM.docx]

Supplementary Table 1. Background of the two groups

|  | >90% LB | <90% LB | P value |
| --- | --- | --- | --- |
| No. of patients | 60 | 39 | - |
| Age (years): mean±SD | 35.32±3.08 | 35.18±2.86 | NS |
| BMI: mean±SD | 20.53±2.17 | 20.19±2.40 | NS |
| Serum AMH (ng/ml): mean±SD | 5.74±8.15 | 3.95±2.88 | NS |
| Duration of infertility (months): mean±SD | 30.15±23.19 | 28.97±26.23 | NS |
| Previous ET: mean±SD | 1.02±1.56 | 1.49±2.04 | NS |
| Multigravida patients: N (%) | 29 (48.3) | 22(56.4) | NS |
| Multipara patients: N (%) | 16 (26.7) | 13 (33.3) | NS |
| Patients with endometriosis: N (%) | 2 (3.3) | 2 (5.1) | NS |
| Patients with myoma: N (%) | 2 (3.3) | 2 (5.1) | NS |
| Patients with PCOS: N (%) | 16 (26.7) | 6 (15.4) | NS |
| Patients with ERA performed: N (%) | 11(18.3) | 6 (15.4) | NS |
| % of endometrial LB: median (range) | 98.95 (90.30-100) | 15.10 (0-87.70) | <.001 |
