# Supplementary material for: Does dysbiotic endometrium affect blastocyst implantation in IVF patients?
Source: J Assist Reprod Genet. 2019 Nov 18;36(12):2471–9. doi: 10.1007/s10815-019-01630-7 (PMC6910901; doi:10.1007/s10815-019-01630-7)
Supplement: Supplementary file 2 — (DOCX 17 kb) [file 10815_2019_1630_MOESM2_ESM.docx]

Supplementary Table 2. Characteristics and pregnancy outcome of two groups

|  | >90% LB | <90% LB | P value |
| --- | --- | --- | --- |
| No. of patients | 60 | 39 | - |
| No. of FBT | 60 | 39 | - |
| No. of transferred blastocysts | 60 | 40 | - |
| Good quality blastocysts transferred: N (%) | 53 (88.3) | 32 (80.0) | NS |
| Laser AHA performed:  N (%) | 44(73.3) | 30 (75.0) | NS |
| No. of pregnancies | 32 | 21 | - |
| No. of ongoing pregnancies* | 24 | 16 | - |
| No. of miscarriages * | 3 | 2 | - |
| No. of twin pregnancies | 0 | 1 | - |
| No. of ectopic pregnancies | 0 | 0 | - |
| Pregnancy rate (per FBT) (%) | 53.3 | 53.8 | NS |
| Implantation rate  (No. of GS/transferred blastocysts) (%) | 53.3 | 55.0 | NS |
| Miscarriage rate (per pregnancy) (%) | 9.4 | 9.5 | NS |
